# Tumor-associated macrophages promote tumor metastasis via the TGF-β/SOX9 axis in non-small cell lung cancer

**DOI:** 10.18632/oncotarget.21068

**Published:** 2017-09-16

**Authors:** Shuai Zhang, Dehai Che, Fang Yang, Chunling Chi, Hongxue Meng, Jing Shen, Li Qi, Fang Liu, Liyan Lv, Yue Li, Qingwei Meng, Junning Liu, Lihua Shang, Yan Yu

**Affiliations:** ^1^ The Sixth Department of Medical Oncology, Harbin Medical University Cancer Hospital, Harbin, China; ^2^ Department of Neurology, The Fourth Affiliated Hospital of Harbin Medical University, Harbin, China; ^3^ Department of Pathology, Harbin Medical University Cancer Hospital, Harbin, China; ^4^ Department of Oncology, The Second Affiliated Hospital of Harbin Medical University, Harbin, China; ^5^ Department of Oncology, The First Affiliated Hospital of Harbin Medical University, Harbin, China; ^6^ Department of Oncology, The First Affiliated Hospital of Qiqihar Medical College, Qiqihar, China

**Keywords:** non-small cell lung cancer (NSCLC), tumor-associated macrophages (TAMs), metastasis, TGF-β, SOX9

## Abstract

Tumor-associated macrophages (TAMs), most of which display the immunosuppressive M2 phenotype, affect the tumor microenvironment and promote progression and metastasis in lung carcinoma. In this study, we analyzed clinical non-small cell lung cancer (NSCLC) samples and found that high densities of TAMs were associated with a poor prognosis in NSCLC patients. Moreover, the number of TAMs present correlated positively with expression of sex determining region Y (SRY)-related high mobility group box 9 (SOX9) in NSCLC tissues. TAMs secreted TGF-β, which increased SOX9 expression and promoted epithelial-to-mesenchymal transition (EMT) in lung cancer cells, thereby promoting tumor proliferation, migration, and invasion. SOX9 knockdown inhibited EMT, indicating that TGF-β-mediated EMT is SOX9-dependent. TGF-β induced SOX9 expression by upregulating the C-jun/SMAD3 pathway. These results indicate that TGF-β secreted by TAMs promotes SOX9 expression via the C-jun/SMAD3 pathway, thereby promoting tumor metastasis. The TGF-β/SOX9 axis may therefore be an effective target for the treatment of lung cancer.

## INTRODUCTION

Lung carcinoma is one of the most commonly diagnosed cancers and the leading cause of cancer-related death, killing more than 1.4 million people worldwide each year [1, 2]. Furthermore, up to 90% of all lung cancer mortality is a result of tumor metastasis [3]. Even after curative resection, many patients remain at risk for local recurrence, distant metastasis, and subsequent development of additional primary tumors [4, 5]. The mechanisms underlying metastasis in lung cancer therefore require further study to improve the accuracy of prognostic predictions.

The development of malignancies and metastasis are closely related to the tumor microenvironment [6]. Macrophages that infiltrate the tumor microenvironment (TME) are considered tumor-associated macrophages (TAMs), and the majority of TAMs display an M2 phenotype [7]. Transforming growth factor-β (TGF-β), a multifunctional cytokine that participates in various biological processes, suppresses cell growth in benign cells but promotes progression in cancer cells [8, 9]. Recent studies have revealed that TAMs release certain factors, such as TGF-β, interleukin-6 (IL-6), and IL-8, that affect the tumor microenvironment [10–12].

Epithelial to mesenchymal transition (EMT) is an important physiological event during mammalian embryo development [13, 14]. Increasing evidence indicates that it is also closely related to the development of epithelial malignancies [15, 16]. In tumors, tumor cells that undergo EMT lose epithelial features and gain mesenchymal phenotypes [17]. EMT allows tumor cells to self-renew and to adapt to different microenvironments [7]. SOX9 is an important gene related to the proliferation and differentiation of cells during early embryonic development [18, 19]. In recent years, research has focused on the role of SOX9 in tumors, and abnormal SOX9 expression occurs in many cancer types [20–22], especially in lung cancer. Sox9 overexpression has been observed in more than 50% of lung adenocarcinomas (ADCs), the most common histological subtype of lung cancer, and is associated with a poor survival in lung ADC patients.

In this study, we examined the role of TAMs in lung carcinoma progression with a focus on TGF-β. We found that TAMs secreted more TGF-β than other macrophage phenotypes. After treatment with supernatant from cultured TAMs, lung carcinoma cells acquired EMT-like properties and expressed EMT markers, and proliferation, migration, and invasion increased in these cells. We also examined clinical specimens and found that TGF-β and SOX9 were highly expressed in cancerous tissues from patients with advanced lung cancer. Furthermore, the number of macrophages that had infiltrated the tumor stroma was correlated with SOX9 expression. Patients with high SOX9 and TGF-β expression had shorter overall survival (OS) than those with low SOX9 and TGF-β expression. We then examined the relationship between TGF-β and SOX9 expression and found that upregulation of TGF-β increased SOX9 expression mainly via the C-jun pathway. In addition, knockdown of SOX9 expression resulted in a loss of the TGF-β-mediated EMT phenotype in tumor cells. Taken together, these results indicate that TAMs promote tumor metastasis via a TGF-β/SOX9 axis in lung carcinoma; this axis might therefore be a target for lung cancer treatments.

## RESULTS

### Number of TAMs is positively correlated with SOX9 expression in human NSCLC tissues

**Figure 1:**
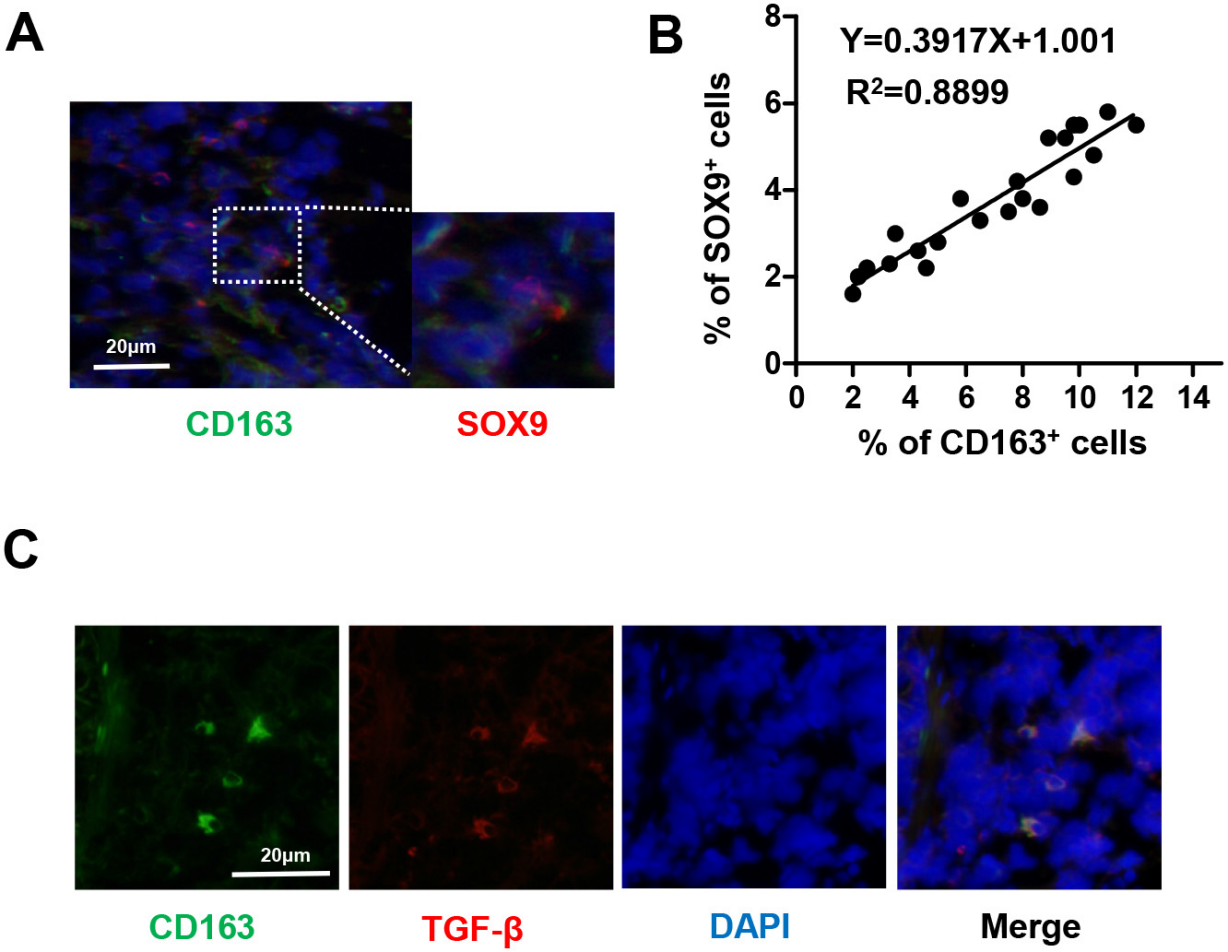
Number of TAMs is correlated with SOX9 expression in NSCLC **(A)** Immunofluorescent staining of human NSCLC tumor tissues. CD163^+^ macrophages are stained green and SOX9^+^ lung cancer cells are stained red. Nuclei were stained with DAPI (blue). **(B)** Linear regression revealed a positive correlation between numbers of CD163^+^ macrophages and SOX9^+^ lung cancer cells. CD163^+^ macrophages and SOX9^+^ lung cancer cells within a distance of 100 μm were counted in 22 separate microscope fields. **(C)** CD163^+^ macrophages express TGF-β; CD163 is stained green, TGF-β red, and nuclei blue.

High densities of tumor-infiltrating TAMs have been found in human lung cancer tissues [23]. TAMs release certain factors, such as TGF-β, interleukin-6 (IL-6), and IL-8, that affect the formation of the tumor microenvironment [24]. Expression of the SOX9 transcription factor has also recently been observed in lung cancer [25]. In addition, TGF-β increased Smad3 phosphorylation and Sox9 reporter luciferase activity [26]. To determine if SOX9 expression is correlated with number of TAMs, we analyzed the distribution of TAMs (CD163^+^ macrophages) and SOX9 in specimens from lung cancer patients using immunofluorescent staining. As shown in (Figure 1A-1B), density of TAMs was positively correlated with SOX9^+^ staining in lung cancer cells In addition, TAMs secreted TGF-β (Figure 1C). These findings suggest that TAMs may promote SOX9 expression.

### Co-expression of CD163 and SOX9 is correlated with worse patient outcomes

**Figure 2:**
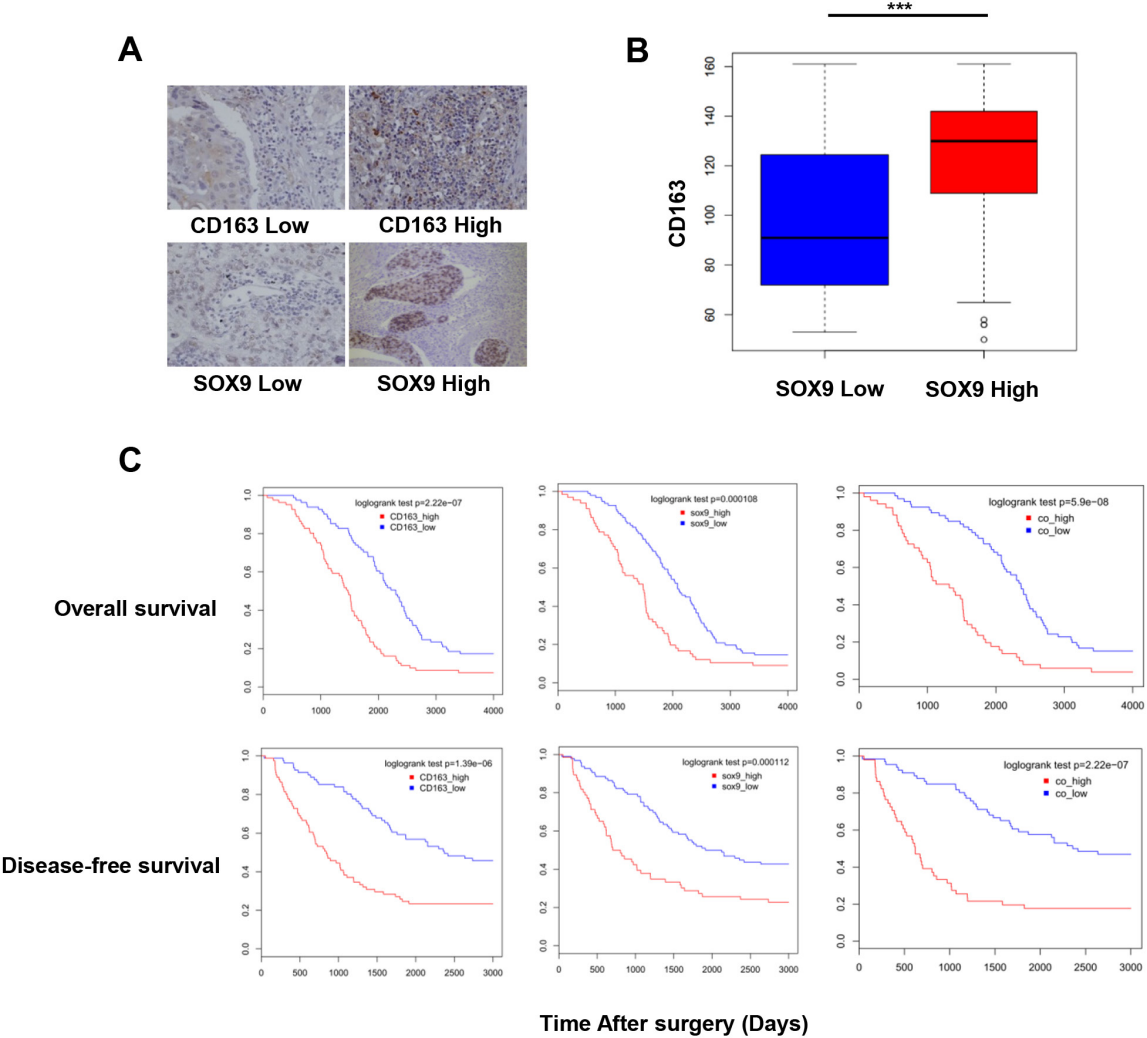
Number of TAMs and SOX9 expression are correlated with patient outcomes **(A)** Immunohistochemistry of human NSCLC tumor tissues. CD163 and SOX9 staining intensities are shown. **(B)** Infiltration of CD163^+^ TAMs was increased in the high SOX9 expression group. **(C)** Overall and disease-free survival in NSCLC patients with high or low CD163 and SOX9 expression were examined. High expression of either CD163 or SOX9 was correlated with lower survival. Patients with high expression of both CD163 and SOX9 had shorter OS and DFS than those with high expression of either CD163 or SOX9. ^***^p < 0.01, mean ± SEM.

Immunohistochemistry revealed that CD163 and SOX9 expression were positively correlated in specimens from 164 lung cancer patients (Figure 2A-2B). CD163 and SOX9 expression were also correlated with disease-free and overall survival in these patients. Patients with high expression of either CD163 or SOX9 had shorter OS and DFS than those with low expression of CD163 or SOX9 (Figure 2C) (*p*<0.01). Furthermore, patients with high expression of both CD163 and SOX9 had shorter OS and DFS than those with high expression of either CD163 or SOX9 alone (Figure 2C) (*p*<0.01). These results indicate that the co-expression of CD163 and SOX9 promotes lung cancer progression and contributes to poorer prognoses.

### Macrophages promote SOX9 expression and transformation into EMT-like phenotype in lung cancer cells

**Figure 3:**
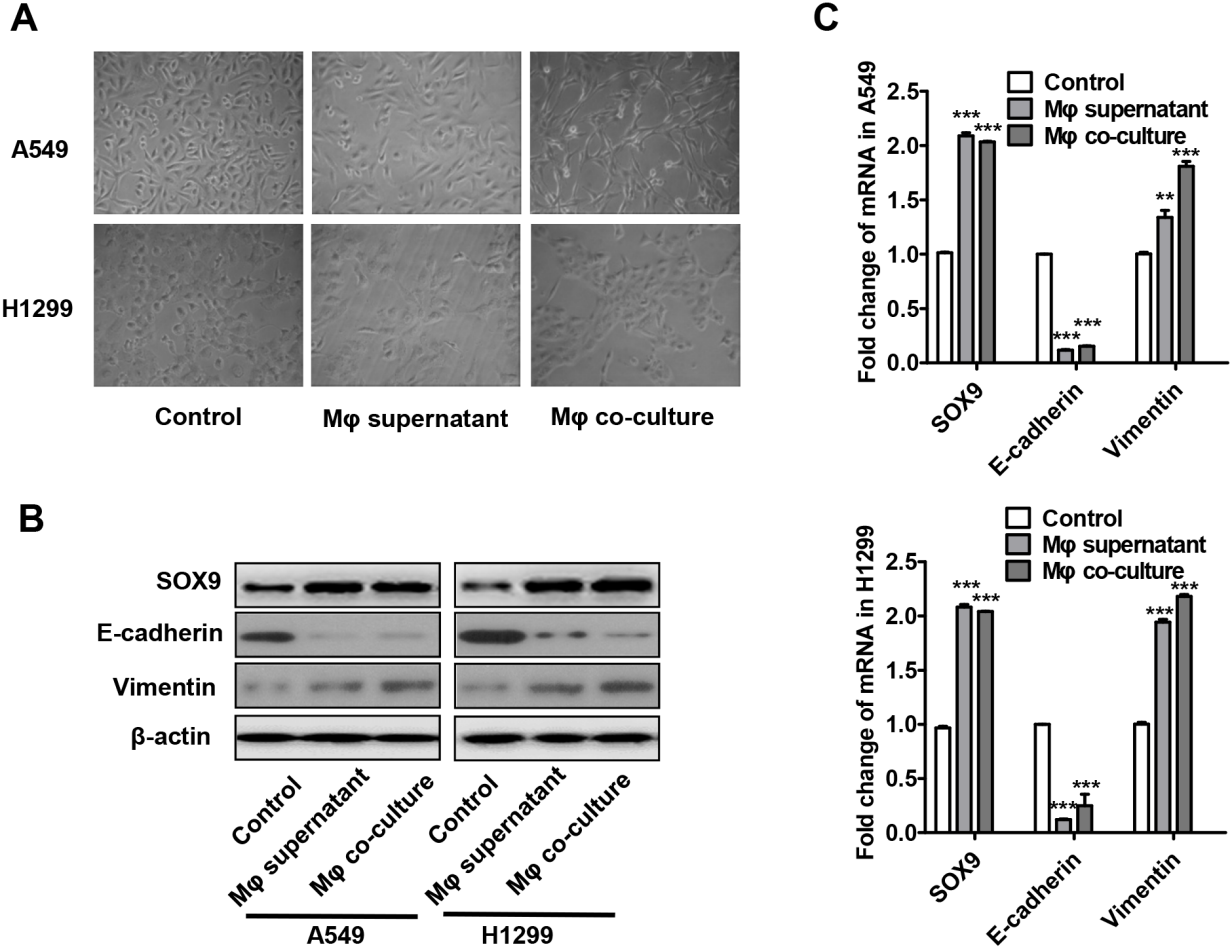
Macrophages promote the transformation of tumor cells into an EMT-like phenotype **(A)** Changes in lung cancer cell morphology after macrophage supernatant was added to A549 and H1299 cells for 48 h or after co-culture of macrophages with A549 and H1299 cells for 48 h. **(B-C)** Changes in SOX9, E-cadherin, and vimentin protein (B) and mRNA (C) levels in lung cancer cells after macrophage supernatant was added to A549 and H1299 cells for 48 h or after co-culture of macrophages with A549 and H1299 cells for 48 h. ^**^p < 0.05, ^***^p < 0.01, mean ± SEM.

Because number of TAMs and SOX9 expression were closely related in lung cancer patients (Table 1 and Figure 1), we hypothesized that TAMs may directly affect SOX9 expression in lung cancer cells. To test this hypothesis, transformation of monocytic THP-1 cells into macrophages was induced as previously described [27]. Supernatant from the macrophage cultures or the macrophages themselves were then added to A549 and H1299 human lung adenocarcinoma cell cultures. After 24 or 48 hours of culture, most A549 and H1299 cells transformed into an EMT-like phenotype (Figure 3A). Changes in the expression of the EMT markers E-cadherin and vimentin were also examined (Figure 3B-3C). Co-culture with either macrophage supernatant or macrophages increased SOX9 protein and mRNA levels in A549 and H1299 cells. (Figure 3B-3C). These results suggest that macrophages promote SOX9 expression and transformation into an EMT-like phenotype in lung cancer cells.

**Table 1:** Characteristics of the 164 NSCLC patients

Variable	Number (N)	Percentage (%)
Gender		
Male	99	60.37
Female	65	39.63
Age		
≤60 years	96	58.54
>60 years	68	41.46
Histological cell type		
Adenocarcinoma	112	68.29
Squamous cell carcinoma	38	23.17
Others	14	8.54
Differentiation		
Well-differentiated	37	22.56
Moderately- or poorly-differentiated	127	77.43
Tumor stage		
I	79	48.17
II	52	31.70
III	33	20.13

### Lung cancer cells induce M2 polarization in macrophages

**Figure 4:**
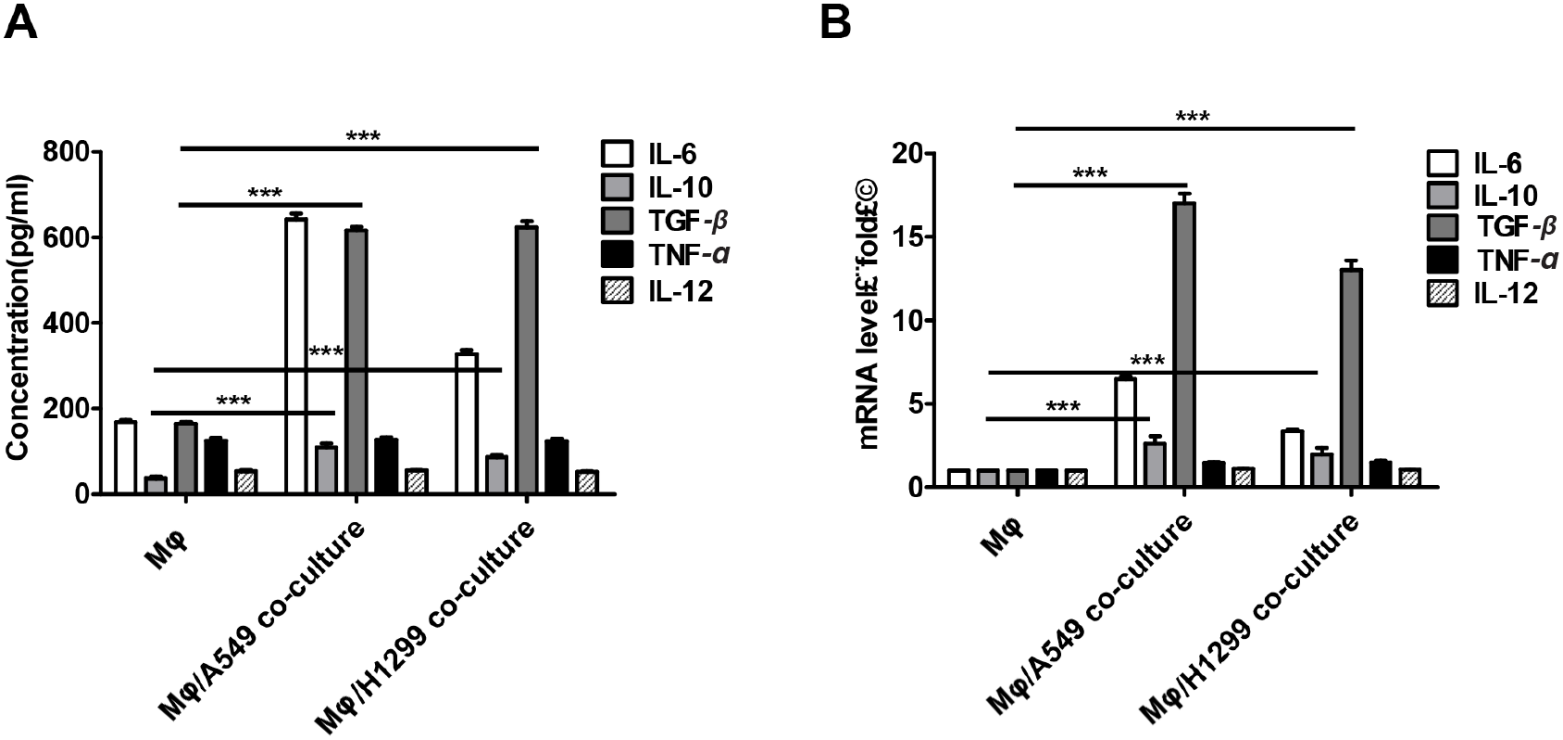
Lung cancer cells induced M2 polarization in macrophages **(A-B)** TGF-β, IL-10, IL-6, and TNF-α protein (A) and mRNA (B) levels in supernatant were determined by ELISA and qRT-PCR, respectively, after incubation with THP-1-derived macrophage supernatants or after co-culture of THP-1-derived macrophages with H1299 or A549 cells for 48 h. ^***^p < 0.01, mean ± SEM.

Interestingly, we also observed that lung cancer cells promoted M2 polarization in macrophages as indicated by changes in levels of the M1-macrophage markers TNF-α and IL-12 and the M2-macrophage markers IL-10 and TGF-β. Higher levels of TGF-β and IL-10 were detected in the supernatant from macrophages co-cultured with A549 and H1299 cells compared to macrophages cultured alone (Figure 4A). We also found that TGF-β and IL-10 mRNA levels were higher in the co-cultured macrophages (Figure 4B). However, no significant differences in TNF-α or IL-12 protein or mRNA levels were observed between the two groups.

### TGF-β increases SOX9 expression and induces transformation into an EMT-like phenotype in lung cancer cells

**Figure 5:**
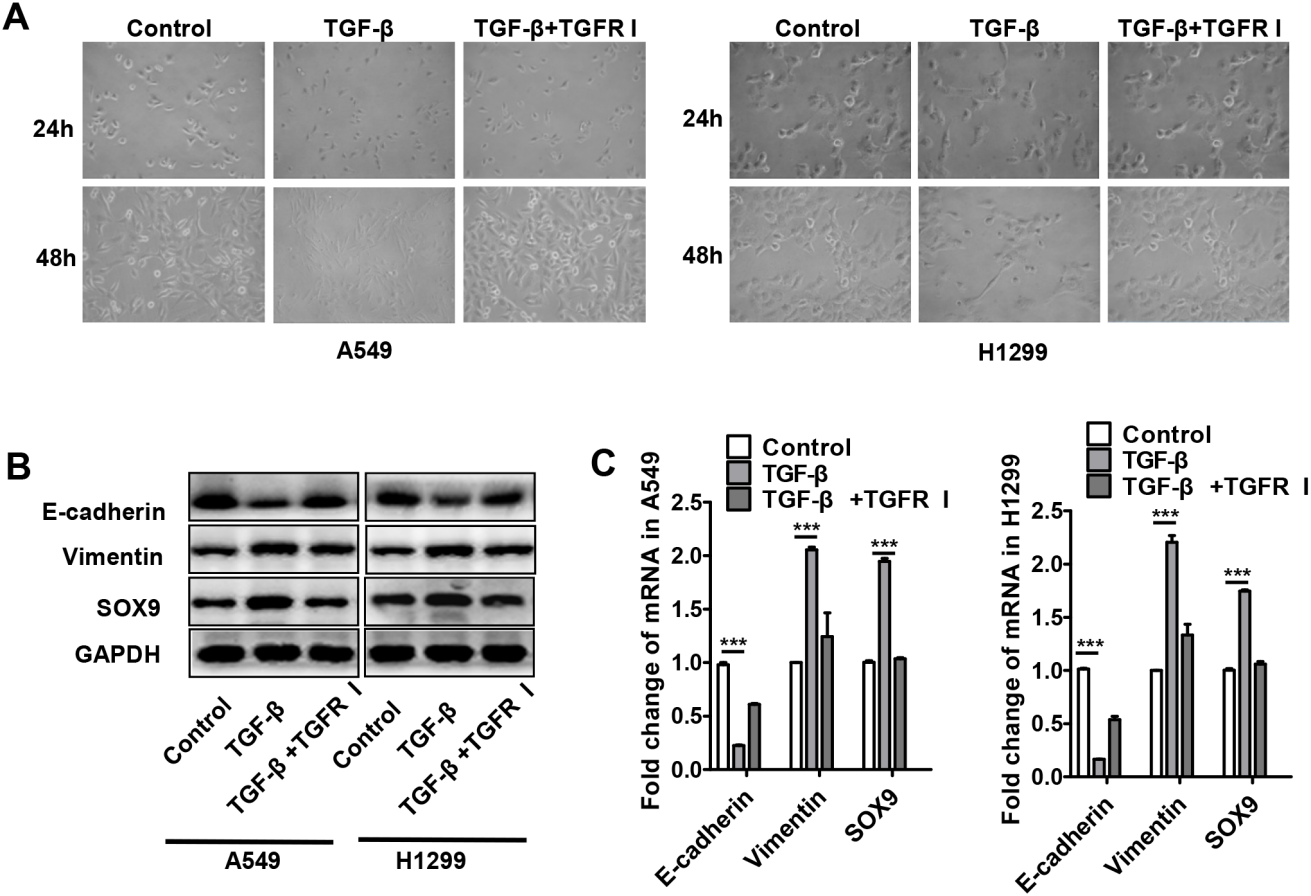
TGF-β increased SOX9 expression and induced transformation into an EMT-like phenotype in lung cancer cells **(A)** Changes in lung cancer cell morphology after recombinant TGF-β (10ng/ml) or TGF-β receptor inhibitor (TGFR I, SD208, 1μM) was added to A549 and H1299 cell culture systems for 24 or 48 h. **(B-C)** Changes in SOX9, E-cadherin, and vimentin protein (B) and mRNA (C) levels in lung cancer cells after recombinant TGF-β or TGFR I was added to A549 and H1299 cell culture systems for 48 h. ^***^p < 0.01, mean ± SEM.

TGF-β is one of the major cytokines secreted by M2 macrophages, and previous experiments revealed that TGF-β-secreting TAMs induced SOX9 expression (Figure 3A-3C). To further examine the effects of TGF-β on SOX9 expression, recombinant TGF-β was added to A549 and H1299 cell cultures. As was observed after co-culture with macrophages, most A549 and H1299 cells transformed into an EMT-like phenotype (Figure 5A), and SOX9 protein levels also increased after 48 h of treatment with recombinant TGF-β (Figure 5B). These changes were dependent on TGF-β signaling and were blocked by a TGF-β receptor inhibitor (Figure 5A-5C). These results indicate that TGF-β promotes the expression of SOX9 and the transformation of tumor cells into an EMT-like phenotype.

### SOX9 plays a key role in TGF-β-mediated induction of EMT-like phenotype in lung cancer cells

**Figure 6:**
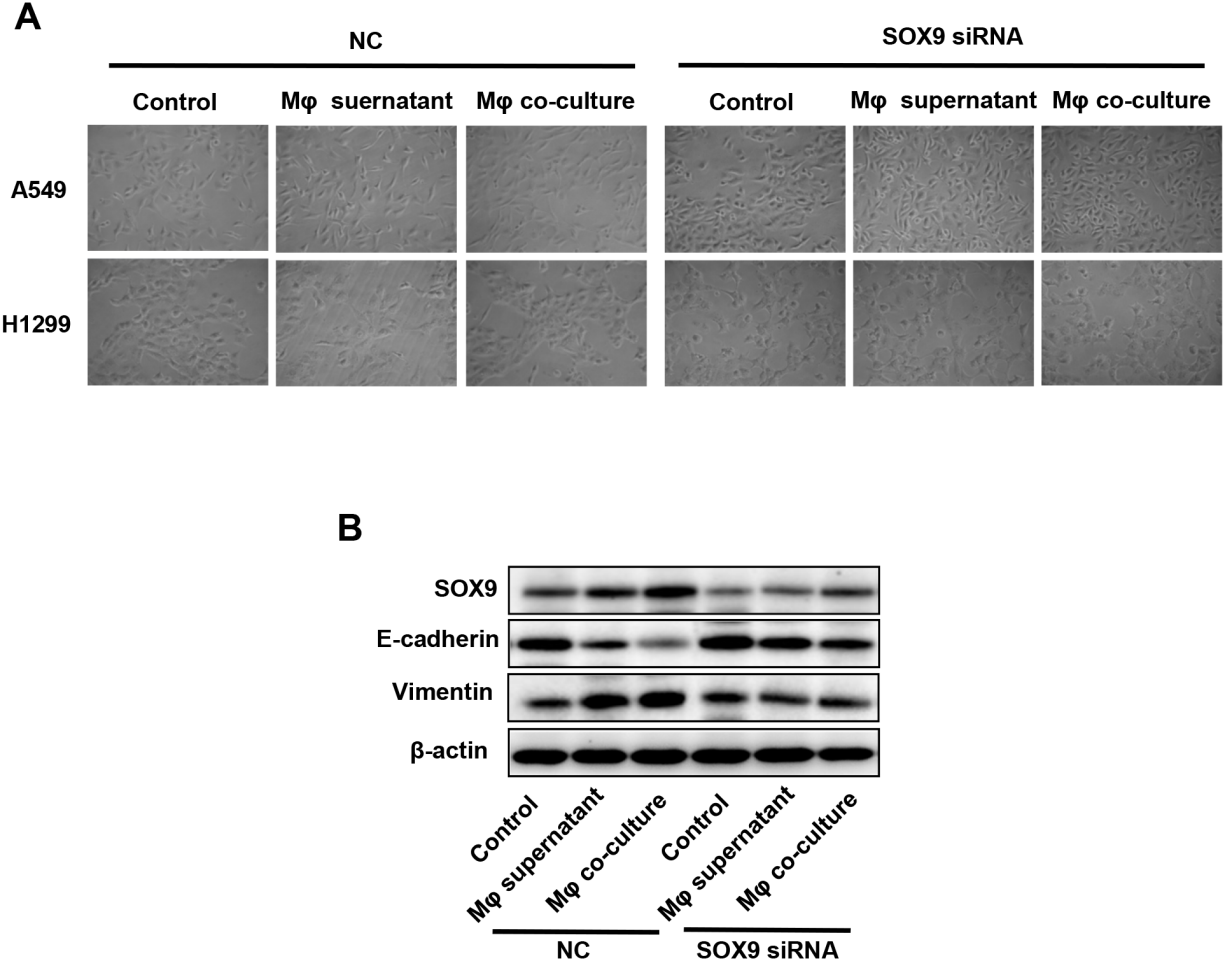
Macrophages induced SOX9-dependent EMT-like phenotype in lung cancer cells SOX9 expression was inhibited in A549 and H1299 cells via RNAi. **(A)** Changes in lung cancer cell morphology after macrophage supernatant was added to A549 and H1299 cells or after co-culture of macrophages with A549 and H1299 cells for 48h. **(B)** Changes in SOX9, E-cadherin, and vimentin protein levels in lung cancer cells after macrophage supernatant was added to A549 and H1299 cells or after co-culture of macrophages with A549 and H1299 cells for 48h.

**Figure 7:**
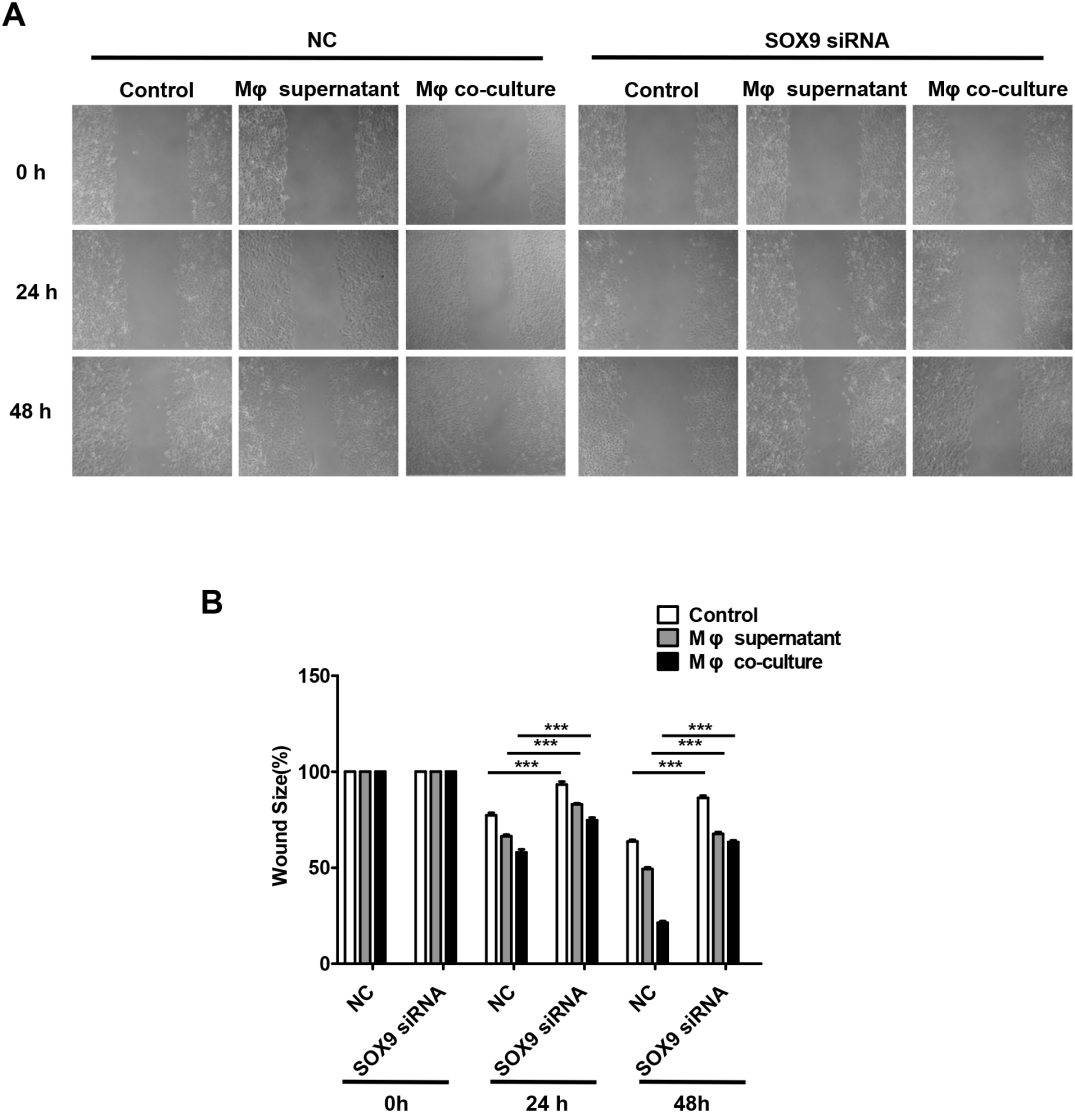
Macrophages promote SOX9-dependent migration in lung cancer cells SOX9 expression was inhibited in A549 cells via RNAi. **(A)** Migration ability was examined in a wound healing assay 48 hours after macrophage supernatant was added to A549 cells or after co-culture of macrophages with A549 cells. **(B)** Mean migration distance of the wound edge after macrophage supernatant was added to A549 cells or after co-culture of macrophages with A549 cells. ^***^p < 0.01, mean ± SEM.

**Figure 8:**
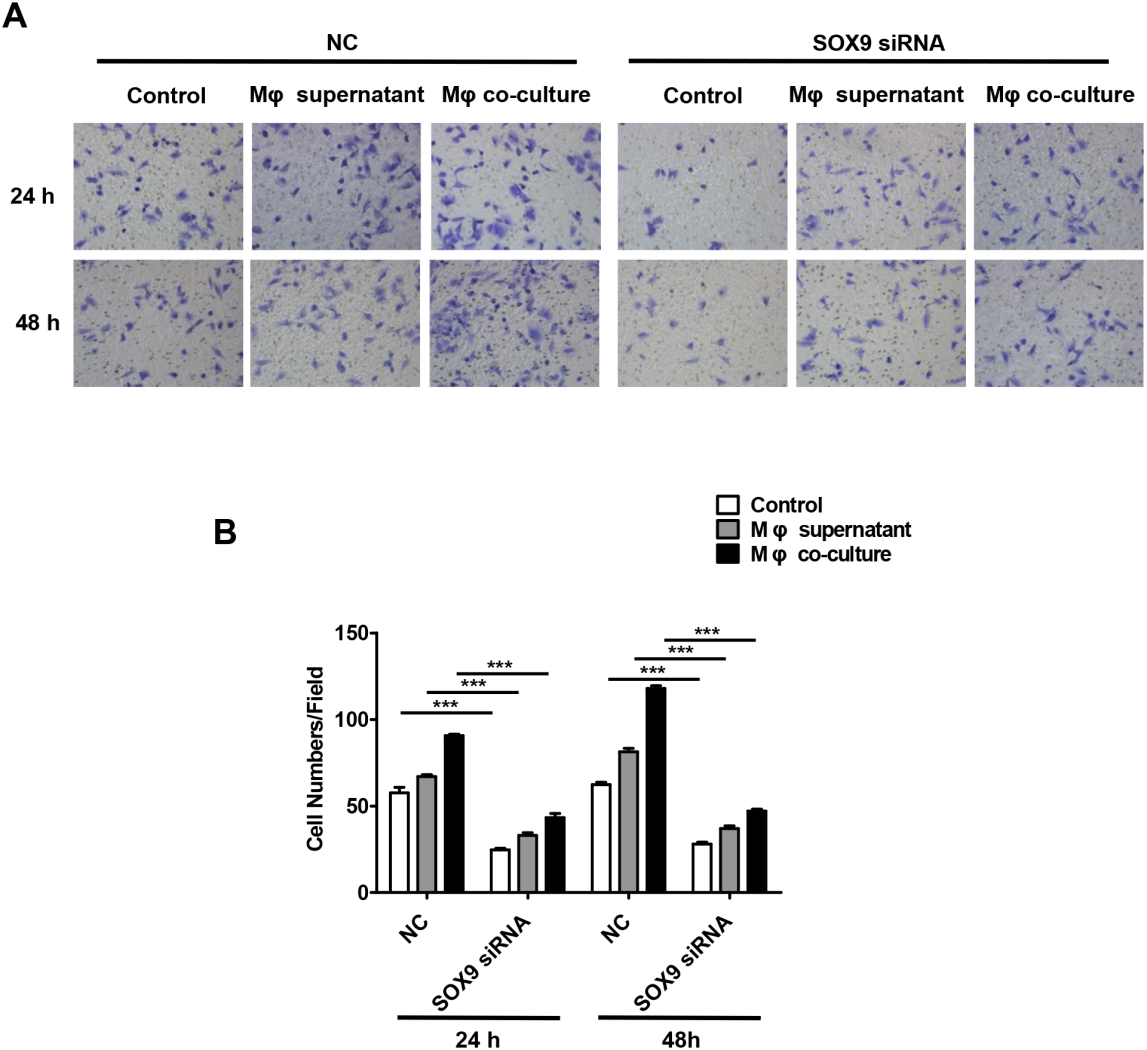
Macrophages promote SOX9-dependent invasion in lung cancer cells SOX9 expression was inhibited in A549 cells via RNAi. **(A)** Invasion assays were performed 48 hours after macrophage supernatant was added to A549 cells or after co-culture of macrophages with A549 cells. **(B)** Total numbers of invaded cells were quantified after macrophage supernatant was added to A549 cells or after co-culture of macrophages with A549 cells. ^***^p < 0.01, mean ± SEM.

**Figure 9:**
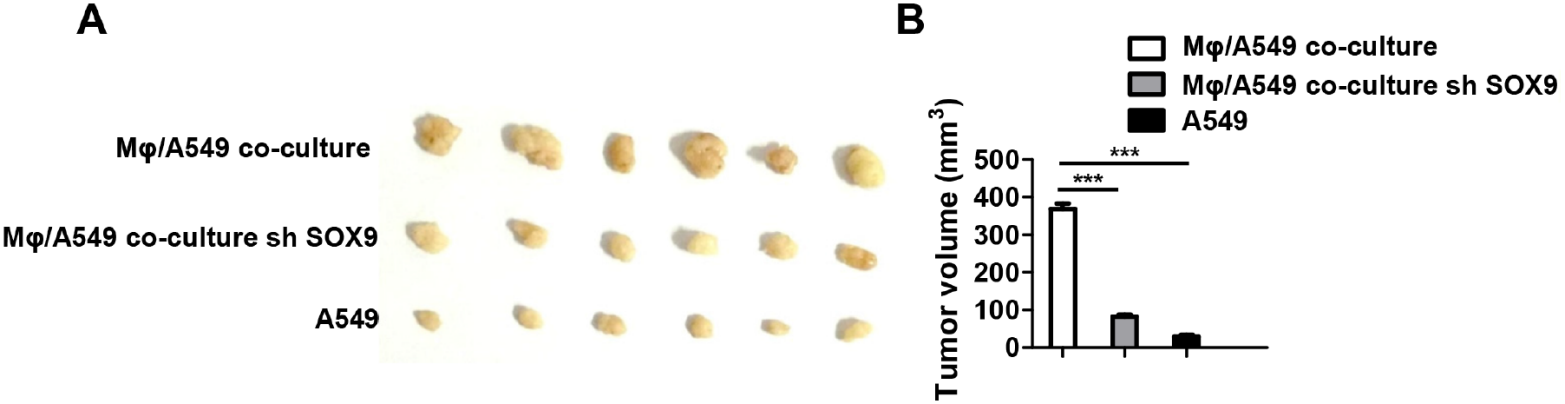
Macrophages promote SOX9-dependent tumorigenicity in lung cancer cells **(A)** Scramble control A549 cells or SOX9-knockdown A549 cells co-cultured with macrophages and control A549 cells cultured alone were subcutaneously injected into mice to form tumors. After three weeks, tumors were harvested and diameters were measured. **(B)** Tumor volumes in the different groups were quantified. ^***^p < 0.01, mean ± SEM.

SOX9 is widely expressed in lung cancer cells and promotes tumor proliferation and metastasis. To further confirm the role of SOX9 in TGF-β-mediated induction of EMT-like phenotype in lung cancer cells, we inhibited SOX9 expression using RNAi in A549 and H1299 cells, which were then co-cultured with macrophages. Examination of cell morphology indicated that EMT was almost completely inhibited and EMT markers E-cadherin and vimentin levels were unchanged in the SOX9 knockdown group compared to the control group (Figure 6A-6B). Tumor cell migration and invasion were also reduced in the SOX9 knockdown group (Figure 7 and Figure 8). To investigate the effect of macrophages or SOX9 on tumor growth *in vivo*, SOX9-knockdown A549 cells co-cultured with macrophages, scramble control A549 co-cultured with macrophages and scramble control A549 cultured alone were injected subcutaneously into nude mice. Measurement of subcutaneous tumor sizes revealed that co-cultured A549 cells with macrophages induced larger tumors than A549 cells cultured alone. Furthermore, SOX9-knockdown A549 cells induced smaller tumors than control A549 cells co-cultured with macrophages (Figure 9A-9B). Taken together, these results indicate that SOX9 plays a key role in macrophage-mediated induction of EMT-like phenotype in lung cancer cells.

### TGF-β promotes SOX9 expression mainly via the C-jun signaling pathway

**Figure 10:**
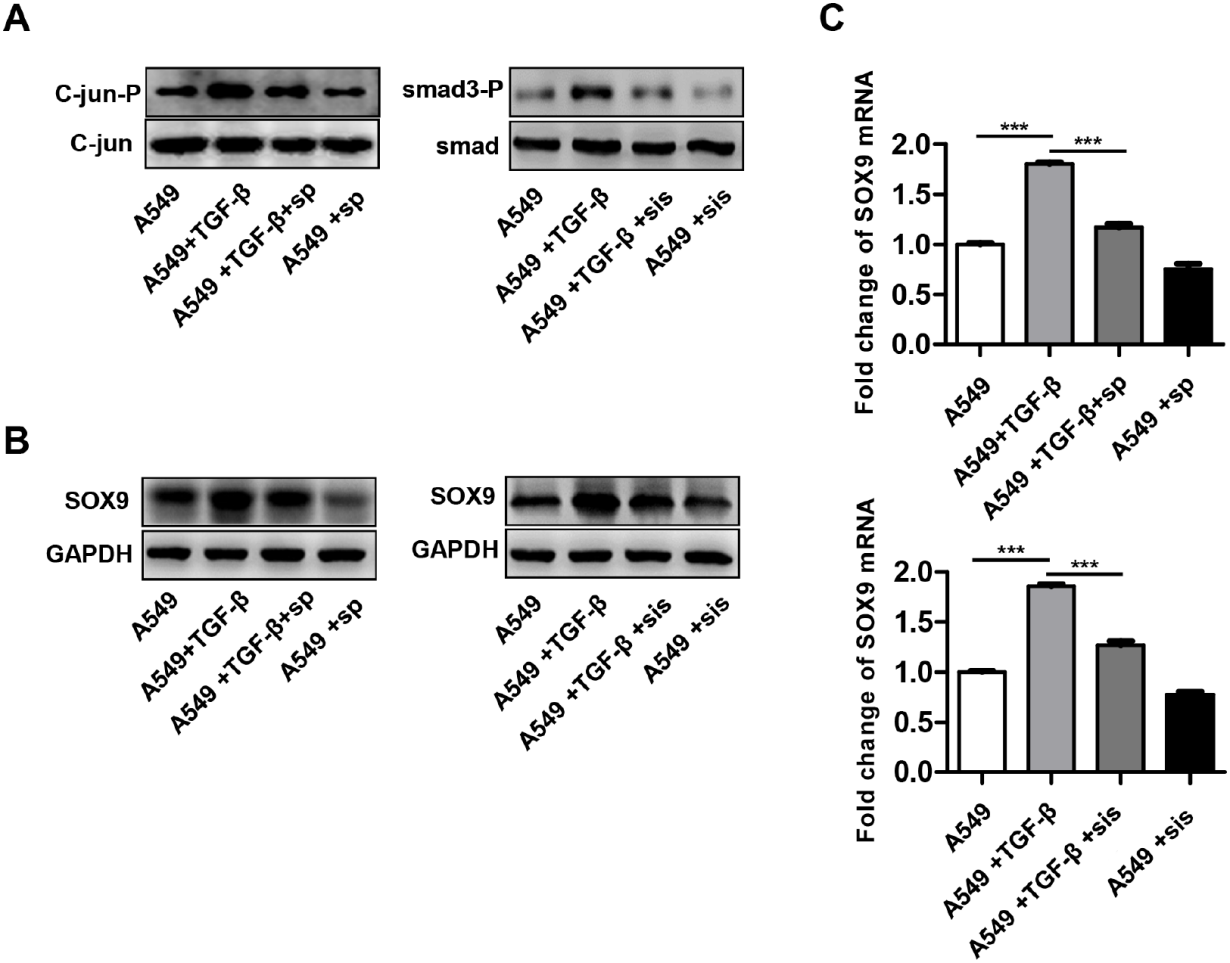
TGF-β promotes SOX9 expression via the C-Jun and SMAD3 signaling pathway A549 cells were pretreated for 0.5 h with a SMAD3 inhibitor SIS3 (sis, 2 μM) or a C-Jun inhibitor SP600125 (sp, 20 μM) and then stimulated with TGF-β (10ng/ml). C-jun and SMAD3 phosphorylation **(A)**, SOX9 protein **(B)** and mRNA levels **(C)** were examined after treatment.

The SMAD signaling pathway is a classical downstream target of TGF-β. Moreover, TGF-β also activated the non-classical C-jun pathway [28]. TGF-β-induced SMAD3 and C-jun phosphorylation was inhibited by administration of either the SMAD3 inhibitor SIS3 or the C-jun inhibitor SP600125 (Figure 10A). Next, we examined the effects of SMAD3 and C-jun inhibitors on SOX9 expression. SOX9 expression decreased 48 h after addition of either SP600125 or SIS3 into the cell culture compared to the control (Figure 10B-10C). Overall, these results indicate that TGF-β promotes SOX9 expression via the C-jun and SMAD3 signaling pathway.

## DISCUSSION

**Figure 11:**
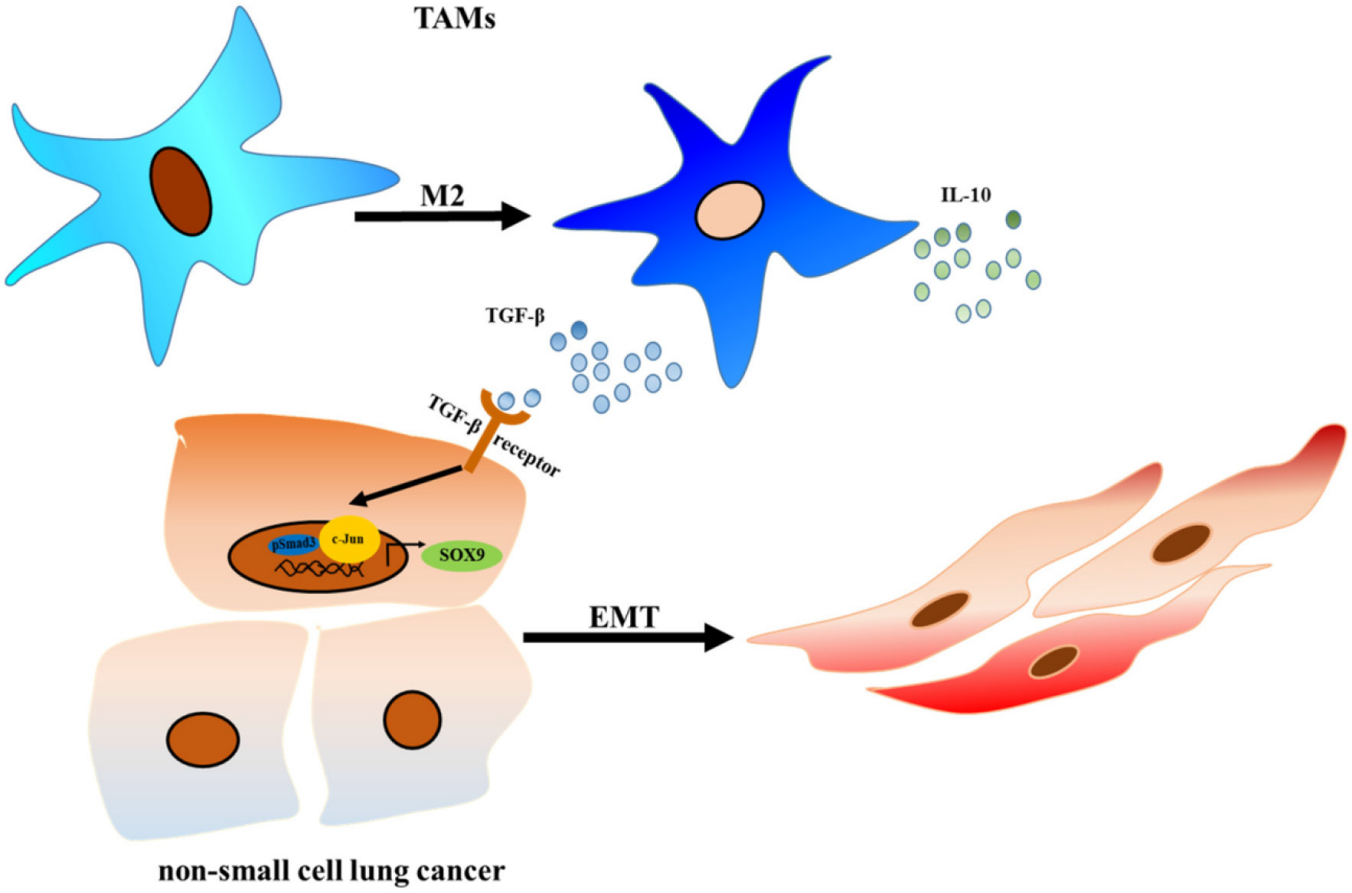
TAMs promote tumor metastasis via the TGF-β/SOX9 axis in non-small cell lung cancer TGF-β secreted by TAMs activated the C-Jun and SMAD3 pathway in lung cancer cells, which increased SOX9 expression and promoted EMT in lung cancer cells. EMT promotes proliferation and metastasis in lung cancer cells.

In 2016, primary lung cancer was one of the most common and deadly human cancers [29, 30]. Epithelial mesenchymal transition (EMT) plays an important role in the metastasis of lung cancer [31], and lung cancer cells undergo EMT when they are co-cultured with macrophages [27]. Although evidence suggests that macrophages promote EMT in lung cancer cells, the underlying molecular mechanisms remain unknown. In this study, we found that lung cancer cells promoted the M2 polarization of tumor-associated macrophages (TAMs), which in turn secretes TGF-β and promotes SOX9 expression via the C-jun/SMAD3 pathway, thereby promoting tumor metastasis (Figure 11).

Macrophages secrete IL-10, TGF-β, IL-6, TNF-α, and many other cytokines [32]. Among these, we found that levels of TGF-β mRNA and protein secreted by macrophages increased the most after co-culture with lung cancer cells (Figure 4). This finding is consistent with previous studies [27]. In addition, Liu *et al.* found that TGF-β promotes EMT in astrocytomas [33]. These results suggest that TGF-β is one of the most important molecules secreted by macrophages that promote EMT in lung cancer cells. SOX9, one of the most widely-studied SOX family transcription factors, is also associated with the development of many human cancers [34–36]. SOX9 expression is abnormally high in lung cancer patients [21], and Capaccione *et al.* found that increased SOX9 expression induced EMT in lung adenocarcinoma cells via Notch1 signaling [37].

We hypothesized that TGF-β induces EMT in lung cancer cells by increasing SOX9 expression. Here, evaluation of 164 patients with lung cancer (Table 1) revealed that number of TAMs was positively correlated with SOX9 expression in lung cancer (Figure 1). In addition, patients with high CD163 or SOX9 expression had shorter overall survival than those with low CD163 or SOX9 expression, respectively. Interestingly, CD163 expression was correlated with SOX9 expression in lung cancer tissues, and patients with high expression of both CD163 and SOX9 exhibited shorter overall and disease-free survival than patients with high expression of either CD163 or SOX9 alone or with low expression of CD163 and SOX9 (Figure 2). We also investigated the relationship between macrophages and SOX9 expression in lung cancer cells. Macrophages promoted SOX9 expression and transformation into an EMT-like phenotype in lung cancer cells (Figure 3 and 6).

The SMAD signaling pathway is a classical downstream target of TGF-β [28]. However, TGF-β also has non-classical and SMAD-independent downstream targets [38]. Here, we found that TGF-β increased phosphorylation of both SMAD3 and C-jun. Surprisingly, C-jun phosphorylation increased to a greater degree than SMAD3 phosphorylation. The addition of either SMAD3 or C-jun inhibitors to the cell cultures inhibited SOX9 expression, and the C-jun inhibitor decreased SOX9 expression to a greater degree than the SMAD3 inhibitor (Figure 9). Furthermore, we found that TAMs and SOX9-positive cells were co-distributed in many NSCLC patients, and these patients had poorer prognoses. Clinical evaluation of TAM populations and SOX9 expression may therefore be an important prognostic indicator in NSCLC. In addition, we found that TGF-β secreted by TAMs increased SOX9 expression and promoted EMT in lung cancer cells by activating the C-Jun pathway. Therapies that target the TGF-β-SOX9 axis to inhibit EMT, which promotes proliferation and metastasis, in lung cancer cells might therefore be effective treatments for NSCLC.

## MATERIALS AND METHODS

### Patient samples

Clinical samples were obtained from 200 NSCLC patients who underwent surgical treatment at Harbin Medical University Cancer Hospital between January 2006 and December 2007. 36 patients withdrew from the study. Follow-up disease assessments were conducted for the remaining 164 patients. None of the patients received any anticancer therapy prior to sample collection. Clinicopathological characteristics for these patients are shown in Table 1. Tumor stage was determined according to the 2010 American Joint Committee on Cancer and International Union against Cancer tumor-node-metastasis (TNM) classification system. Tumor differentiation was graded according to the Edmondson and Steiner grading system. All experiments were performed in accordance with the relevant guidelines and regulations of Harbin Medical University, and the study was approved by the Ethics Committee of Harbin Medical University; written informed consent was obtained from each patient.

### Immunohistochemistry and assessment

Surgical specimens were embedded in paraffin and then sliced into 6 μm-thick sections. Anti-human SOX9 (1:200; ab185966; Abcam) and CD163 (1:200; ab17051; Abcam) antibodies were used for immunohistochemical staining; CD163 is a marker of M2-type tumor-associated macrophages (M2-TAMs). Images were taken with a light microscope (Zeiss, Germany). Stained sections were screened at 100× magnification to identify the regions with the highest numbers of TAMs as indicated by immunohistochemical staining of CD163. Analysis of immunostaining was performed independently by two researchers, one of whom was a pathologist, who were blind with respect to clinical outcomes. Images of five separate fields were taken for each sample; cells positive for CD163 staining were considered TAMs and counted. Counts generated by the two researchers were compared to assess the reproducibility and validity of the results. The median number of TAMs per field under 200× magnification was 118; this value was chosen as the cut-off level for distinguishing samples with high and low TAMs counts. Representative pictures of low and high TAM counts (CD163 expression) are shown in Figure 2A. Sox9 expression was assessed based on the intensity of nuclear and/or cytoplasmic anti-SOX9 immunostaining, which was assigned scores of 0 (no staining) to 3 (strongest intensity). In brief, scores were assigned as follows: 0 indicated negative staining or positive staining in <10% of tumor cells; 1 indicated positive staining in 11% to 50% of cells; 2 indicated positive staining in 51% to 80% of cells; and 3 indicated positive staining in >80% of cells. Scores of 0 and 1 were defined as low SOX9 expression, and scores of 2 and 3 were defined as high SOX9 expression.

### Immunofluorescence

Fresh surgical specimens were embedded in optimal cutting temperature compound. Seven μm-thick cryosections were air-dried, fixed for 10 min at room temperature using 4% paraformaldehyde, and then blocked with 0.5% bovine serum albumin (BSA) for 1 h to prevent non-specific binding. Sections were then incubated with anti-SOX9 (1:200; ab185966; Abcam), anti-CD163 (1:200; ab17051; Abcam), and anti-TGF-β (1:200; bs-0086R) antibodies for 12 h at 4°C. After the sections were washed with PBS, they were incubated with goat anti-rabbit IgG-FITC (sc-2012, Santa Cruz Biotechnology) and mouse anti-rabbit IgG-PE (sc-3753 Santa Cruz Biotechnology) for 2 h at 37°C, followed by an additional PBS wash. Slides were stained with DAPI (sc-3598, Santa Cruz Biotechnology) for 2 min. After washing in PBS, coverslips were mounted onto the slides using anti-fade reagent (P36930, Life Technologies). Images were acquired using a fluorescent microscope (Leica TCS SP2). To calculate percentages of cells that were CD163-, TGF-β-, and SOX9-positive, the number of cells with positive staining and the total number of cells were counted in five count areas for each photograph using Image-Pro Plus v6.0 (Media Cybernetics, Inc.). The total positive cell number was divided by the total number of cells to obtain the mean percentage of positive cells. Counts were performed by two observers, and the mean value was used for analysis.

### Cell lines and cell culture

The H1299 and A549 human lung adenocarcinoma cell lines and the THP-1 monocyte cell line were purchased from American Type Cell Collection (ATCC, Manassas, VA, USA). The cells were cultured in RPMI-1640 medium supplemented with 10% fetal bovine serum (Gibco, USA), 100 μg/mL streptomycin, and 100 units/mL penicillin (Invitrogen, Carlsbad, CA, USA). Cells were maintained at 37ºC in a humidified 5% CO_2_ atmosphere. In the experiments, A549 and H1299 cells were incubated with 10 ng/mL of recombinant human TGF-β (100-21C, PeproTech) in RPMI-1640 medium for 48 h. To stimulate transformation of THP-1 cells into macrophages, 50 ng/mL PMA (Sigma Chemical) were added to the medium for 48 h; cells were then washed three times with PBS and incubated for another 24 h in the absence of PMA.

### Co-culture procedures

THP-1 derived macrophages and lung cancer cells were co-cultured using a cell culture insert (Corning, NY, USA) with a porous membrane to separate the upper and lower chambers. The macrophages (7.5×10^5^ cells/well) were seeded into the upper chamber of the transwell apparatus, and the A549 and H1299 cells were placed in the lower chamber (3×10^5^ cells/well). The chambers with the THP-1-derived macrophages were then placed directly on top of six-well plates containing the A549 and H1299 cells, and the resulting co-culture systems were incubated for 24 h or 48 h in serum-free RPMI 1640. Lung cancer cells and THP-1-derived macrophages were cultured for 24 h in serum-free RPMI 1640 as controls. To study the role of TGF-β, anti-TGF-β antibody (R&D Systems, Minneapolis, MN, USA) was added to the co-culture system.

### Quantitative real-time PCR

Total RNA was extracted from THP-1-derived macrophages and lung cancer cells cultured alone and from lung cancer cells co-cultured with THP-1-derived macrophages using Trizol reagent (No. 51–0700, Invitrogen, Camarillo, CA, USA) according to the manufacturer’s protocol. DNA was then reverse transcribed using Moloney murine leukemia virus reverse transcriptase (51–0700, Invitrogen). Quantitative PCR was performed according to the manufacturer’s instructions using SYBR Premix Ex Taq (Takara, Japan). For analysis, the expression of target genes was normalized to Glyceraldehyde 3-phosphate dehydrogenase (GAPDH) expression. All primers were synthesized by Sangon Biotech (China) and are shown in Table 2.

**Table 2:** Primer sequences used for QRT-PCR

Name	Forward	Reverse
SOX-9	5’- GACTTCTGAACGAGAGCGAGA-3’	5’-CGTTCTTCACCGACTTCCTC-3’
GAPDH	5’-TTCGACAGTCAGCCGCATCTTCTT-3’	5’-GCCCAATACGACCAAATCCGTTGA-3’
E-cadherin	5’-TTGCTCACATTTCCCAACTCCTC-3’	5’-CACCTTCAGCCATCCTGTTTCTC-3’
Vimentin	5’-GCTGAATGACCGCTTCGCCAAC-3’	5’-AGCTCCCGCATCTCCTCCTCGTA-3’
TGF-β	5’- TGTACCGCTATGGTTACACTCG-3’	5’- GGCAGGGACAGTTGCTTCT-3’
IL-10	5’-GACTTTAAGGGTTACCTGGGTTG-3’	5’-TCACATGCGCCTTGATGTCTG-3’
IL-6	5’-TCATCACTGGTCTTTTGGAG-3’	5’-GTCAGGGGTGGTTATTGC--3’
TNF-α	5’-CTACTCCCAGGTTCTCTTCAA-3’	5’-GCAGAGAGGAGGTTGACTTTC-3’

### Western blotting

Cells were lysed with lysis buffer (Cell Signaling Technology, Danvers, USA) containing a protease inhibitor (Sigma Chemical company, St. Louis, USA). Protein concentration was quantified using a BCA protein assay kit (Santa Cruz, USA). Western blotting was performed as previously described [39]. Specific primary antibodies against p-Smad3 (1:1000, ab52903, Abcam), c-jun (1:1000, ab321137, Abcam), Smad3 (1:1000, ab40854, Abcam), p-c-jun (1:1000, ab32385, Abcam), SOX9 (1:1000, ab185230, Abcam), E-cadherin, Vimentin, GAPDH (1:1000, 2118s, CST), and β-actin and secondary mouse anti-rabbit IgG-HRP (1:3000, 5127, CST) and goat anti-mouse IgG-HRP (1:5000, Santa Cruz Biotechnology, Santa Cruz, CA) antibodies were used.

### ELISA for cytokine detection

Supernatants from the THP-1-derived macrophages and from the H1299 and A549 cells cultured alone were collected after incubation in serum-free RPMI for 48 h. The medium was centrifuged to remove cellular debris and then frozen at -80ºC until cytokine levels were assayed. TGF-β, IL-10, IL-6, and TNF-α levels were measured using an ELISA kit (Uscn Life Science, Houston, USA) according to the manufacturer’s instructions.

### Invasion assays

Invasion assays were conducted using a 24-well transwell chamber with a polycarbonate membrane with a pore size of 8 μm (Corning, NY, USA). The membrane was coated with 60 μL of a 1:3 mixture of matrigel (BD Sciences, San Jose, CA, USA) and serum-free RPMI 1640 medium to form a matrix barrier. After the matrigel was allowed to solidify at 37ºC for 2 h, lung cancer cells from each group (1×10^5^ cells/mL) were added to the upper compartment of the chamber. The lower chamber was filled with 0.6 mL of medium supplemented with 10% FBS as a chemoattractant. After incubation for 24 h or 48 h, cells that migrated to the lower surface of the membrane were fixed with 4% paraformaldehyde and stained with 0.5% crystal violet. Migrated cell numbers were counted in five random fields at 200× magnification and averaged.

### Wound-healing assays

Cells were seeded in a 6-well plate and allowed to grow to nearly 100% confluence in culture medium. Subsequently, a cell-free line was manually created by scratching the confluent cell monolayers with a 200 μL pipette tip. The wounded cell monolayers were washed three times with PBS and incubated in RPMI 1640 with 10% FBS for 48 hours after macrophage supernatant was added or after co-culture of macrophages. Images of five randomly-selected scratched fields were captured using a bright-field microscope (IX51; Olympus). Wound closure percentage was measured using Adobe Photoshop CS2 (Adobe Systems Inc., San Jose, CA, USA). The experiment was performed independently three times to generate triplicate measurements.

### Transient transfection with siRNA

For siRNA transfection, SOX9-siRNA (GGAGACUUCUGAACGAGAGTT, CGCUCA CAGUACGACUACATT, GCGAAAUCAACGA GAAACUTT) and negative control RNA (UUCUCCGAACGUGUCACGUTT) were synthesized by Oligofectamine (Invitrogen, San Diego, CA). NSCLC cells were plated in 6-well plates at a density of 3 × 10^5^ cells per well and then transfected with 60 nM negative control siRNA or 20 nM each of the three SOX9 siRNAs using the Lipofectamine™ 2000 Transfection Reagent according to the manufacturer's instructions (Invitrogen).

### Xenograft models

Healthy purebred BALB/C nude mice (6 weeks old) were maintained according to the guidelines for the administration of laboratory animal research as outlined by the Institutional Animal Care and Use Committee of Harbin Medical University in China and the Care and Use of Laboratory Animals (National Institutes of Health, revised 1985). To assess the effect of macrophages or SOX9 on tumor growth, A549 control, shSOX9-A549/macrophage-treated, or A549/macrophage-treated cells (5×10^6^ cells in 100μL of PBS) were injected into the ventral regions of six female BALB/C nude mice. Tumor growth was monitored weekly by measuring perpendicular tumor diameters (length (L) and width (W)) with a Vernier caliper. Tumor volume (V) was calculated using the formula V = LW^2^/2. The research protocol was approved by the institutional ethics committee for the administration of laboratory animals of Harbin Medical University, China.

### Statistics

Statistical analysis was performed with SPSS 17.0 software (SPSS, Chicago, IL). Each treatment was performed in duplicate and all experiments were independently repeated three times. Measurement values are expressed as means ± standard deviations. Student’s *t* test and Spearman’s *r* correlation were used as appropriate to assess statistically significant differences. Survival rates were evaluated using the Kaplan-Meier method (log-rank test). *P* < 0.05 indicated statistically significant results.

## Abbreviations

TAMs: Tumor-associated macrophages
NSCLC: non-small cell lung cancer
SRY: sex determining region Y
TME: tumor microenvironment
TGF-β: Transforming growth factor-β
EMT: Epithelial to mesenchymal transition
SOX9: sex determining region Y-related high mobility group box 9
TNM: tumor-node-metastasis
EMT: Epithelial mesenchymal transition
