# Correction: Tumor-associated macrophages promote tumor metastasis via the TGF-β/SOX9 axis in non-small cell lung cancer

**DOI:** 10.18632/oncotarget.27740

**Published:** 2020-12-29

**Authors:** Shuai Zhang, Dehai Che, Fang Yang, Chunling Chi, Hongxue Meng, Jing Shen, Li Qi, Fang Liu, Liyan Lv, Yue Li, Qingwei Meng, Junning Liu, Lihua Shang, Yan Yu

**Affiliations:** ^1^ The Sixth Department of Medical Oncology, Harbin Medical University Cancer Hospital, Harbin, China; ^2^ Department of Neurology, The Fourth Affiliated Hospital of Harbin Medical University, Harbin, China; ^3^ Department of Pathology, Harbin Medical University Cancer Hospital, Harbin, China; ^4^ Department of Oncology, The Second Affiliated Hospital of Harbin Medical University, Harbin, China; ^5^ Department of Oncology, The First Affiliated Hospital of Harbin Medical University, Harbin, China; ^6^ Department of Oncology, The First Affiliated Hospital of Qiqihar Medical College, Qiqihar, China

**This article has been corrected:** Due to errors in image selection, the pictures of H1299 cell morphology treated with recombinant TGF-β and TGF-β receptor inhibitor in Figure 5A are incorrect. When preparing the results, the control group data was mistakenly copied and presented as the treatment group. The corrected Figure 5 is shown below. The authors declare that these corrections do not change the results or conclusions of this paper.

Original article: Oncotarget. 2017; 8:99801–99815. 99801-99815. https://doi.org/10.18632/oncotarget.21068

**Figure 5:**
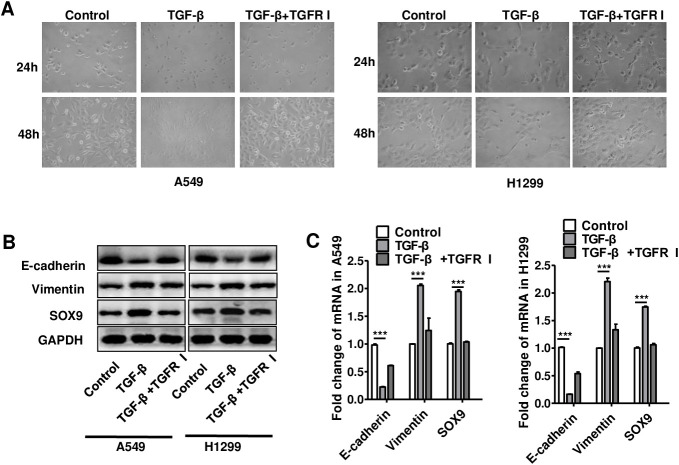
TGF-β increased SOX9 expression and induced transformation into an EMT-like phenotype in lung cancer cells. (**A**) Changes in lung cancer cell morphology after recombinant TGF-β (10ng/ml) or TGF-β receptor inhibitor (TGFR I, SD208, 1μM) was added to A549 and H1299 cell culture systems for 24 or 48 h. (**B**–**C**) Changes in SOX9, E-cadherin, and vimentin protein (B) and mRNA (C) levels in lung cancer cells after recombinant TGF-β or TGFR I was added to A549 and H1299 cell culture systems for 48 h. ^***^ *p* < 0.01, mean ± SEM.

